# Assessment and Comparison of Hepatitis C Viremia in the Prison Systems of New Mexico and Georgia

**DOI:** 10.1001/jamanetworkopen.2019.10900

**Published:** 2019-09-06

**Authors:** Anne C. Spaulding, Junyu Chen, Carolyn A. Mackey, Madeline G. Adee, Chava J. Bowden, W. David Selvage, Karla A. Thornton

**Affiliations:** 1 Rollins School of Public Health, Emory University, Atlanta, Georgia; 2 New Mexico Corrections Department, Santa Fe; 3 University of New Mexico Health Sciences Center, Albuquerque

## Abstract

This cross-sectional study assesses and compares the prevalence of hepatitis C virus among criminal justice populations in New Mexico, a state with high hepatitis C virus prevalence, and Georgia, a state with low hepatitis C virus prevalence.

## Introduction

Recently published estimates by Rosenberg et al^1^ of hepatitis C virus (HCV)–infected persons by state, using National Health and Nutrition Examination Survey (NHANES) data for community-dwelling persons, included an estimate of the prevalence of viremia of 10.7% among all criminal justice (CJ) populations.^1,2^ Although those estimates included a sensitivity analysis varying the non-NHANES prevalence rates (mostly among the CJ population, and to a lesser extent homeless individuals) to mirror statewide epidemics,^1^ we are concerned that failure to consider other data sources^3,4^ may have led to an artificial flattening of the peaks and valleys of statewide HCV estimates.

National surveys of HCV antibody prevalence (seroprevalence) in state prison systems consistently show heterogeneity, varying 5- or 6-fold by state, with New Mexico having the highest seroprevalence of HCV.^4^ Injection drug use, the most common risk factor for HCV, is relatively frequent there. The New Mexico Corrections Department (NMCD) has conducted universal HCV antibody screening at intake since 2009 and reflex HCV RNA testing since 2017. Georgia has less injection drug use, a higher incarceration rate, and a higher degree of disproportional minority confinement.^5^ This cross-sectional study assesses and compares the prevalence of HCV among CJ populations in NMCD and the Georgia Department of Corrections (GDC).

## Methods

We conducted a cross-sectional study of antibody and viremia prevalence from NMCD entry testing and GDC exit testing. We followed Strengthening the Reporting of Observational Studies in Epidemiology (STROBE) reporting guidelines. The Emory University institutional review board determined that each state’s surveillance constitutes public health practice rather than human subjects research. Nonetheless, GDC required specific, written informed consent for HCV testing. No such requirement exists in NMDC.

We evaluated age, HCV seroprevalence, and viremia of persons entering NMCD in 2018, compared with those tested on release from 6 GDC facilities from October 2016 through May 2018. The New Mexico Corrections Department sent specimens to BioReference Laboratories; GDC sent specimens to Quest Diagnostic Laboratories. Antibody testing was performed using Centaur (Siemens Healthineers) for specimens from NMCD and test kits from Ortho Clinical Diagnostics for specimens from GDC. Both states used COBAS AmpliPrep/COBAS TaqMan HCV Test version 2.0 (Roche) for reflex testing of seropositive specimens; the lower limit of detection of HCV is 15 IU/mL.

Logistic regression was performed to calculate odds ratios (ORs) and 95% CIs for viremia by birth cohort from 1965 and earlier vs subsequent birth cohorts for men in NMCD and for men and women in GDC. We used 2-tailed χ^2^ tests (α = .05) to assess statistical significance of age as a categorical variable. Analyses were performed using SAS version 9.4 statistical software (SAS Institute, Inc).

## Results

Table 1 shows HCV testing results for antibodies and viremia by birth cohort. The HCV viremia prevalence among male and female entrants in NMCD was 40.8% (95% CI, 39.3%-42.5%). In this predominantly male (89.4%) prison system, HCV prevalence among men was 42.6% (95% CI, 40.9%-44.3%). The HCV viremia prevalence among tested persons exiting GDC was 6.1% (95% CI, 4.2%-8.6%). For men in NMCD, the OR for viremia by birth cohort from 1965 and earlier vs all others was 0.50 (95% CI, 0.36-0.69; *P* < .001). For GDC, this OR was 3.00 (95% CI, 1.20-6.87; *P* = .01).

**Table 1.** Hepatitis C Virus Seroprevalence and Viremia Among Entrants to NMCD and GDC by Birth Cohort

Birth Cohort	NMCD^a^					GDC^b^				
	Tested, No.	HCV Ab Positive, No. (%)	HCV RNA Positive, No. (%)	HCV Viremia		Tested, No.	HCV Ab Positive, No. (%)	HCV RNA Positive, No. (%)	HCV Viremia	
				OR (95% CI)	*P* Value				OR (95% CI)	*P* Value
Total	3295	1688 (51.1)	1405 (42.6)			494	48 (9.7)	30 (6.1)		
1965 and earlier	191	79 (41.4)	51 (26.7)	1 [Reference]	NA	58	14 (24.1)	8 (13.8)	1 [Reference]	NA
1966-1975	450	233 (51.8)	191 (42.4)	0.50 (0.36-0.69)	<.001	96	9 (9.4)	7 (7.3)	3.00 (1.20-6.87)	.01
1976-1985	1043	529 (50.7)	435 (41.7)			153	14 (9.2)	9 (5.9)		
1986-1995	1348	752 (55.8)	631 (46.8)			170	10 (5.9)	6 (3.5)		
After 1995	248	86 (34.6)	85 (34.3)			16	1 (6.3)	0		
Not recorded	15	9 (60.0)	12 (80.0)			1	0	0		

Abbreviations: Ab, antibody; GDC, Georgia Department of Corrections; HCV, hepatitis C virus; NA, not applicable; NMCD, New Mexico Corrections Department; OR, odds ratio.

^a^ Birth cohorts comprise men entering NMCD in 2018.

^b^ Birth cohorts comprise men and women released from 6 GDC facilities from October 2016 through May 2018 who accepted HCV testing.

Rosenberg et al^1^ estimated 26 700 cases of HCV statewide for New Mexico. However, when we substituted observed NMCD values for overlapping non-NHANES populations, the statewide prevalence in New Mexico increased to 33 521 cases (Table 2). When we substituted the observed HCV prevalence from GDC, the number of cases decreased to 26 274. The absolute difference using NMCD vs GDC data was 7247 cases. The same exercise for Georgia showed that the estimate by Rosenberg et al^1^ of 56 800 cases of HCV decreased to 54 425 using the observed prevalence from GDC and increased to 100 092 cases using the observed prevalence from NMCD, with an absolute difference of 45 667 cases.

**Table 2.** Sensitivity Analysis for Substituting Natural Variations of HCV Prevalence in Criminal Justice Populations Into Non–National Health and Nutrition Examination Survey Portion of Model by Rosenberg et al^1^

Model	Statewide Estimated No. of HCV Cases^a^	
	New Mexico	Georgia
Model by Rosenberg et al^1^	26 700	56 800
If NMCD observed prevalence of HCV used	33 521	100 092
If GDC observed prevalence of HCV used	26 274	54 425
Absolute difference between using NMCD and GDC observed prevalence of HCV	7247	45 667

Abbreviations: GDC, Georgia Department of Corrections; HCV, hepatitis C virus; NMCD, New Mexico Corrections Department.

^a^ For men and women in 2018.

## Discussion

We found that substituting natural variations in the prevalence of HCV among CJ populations into the non-NHANES portion of the model by Rosenberg et al^1^ changed the overall statewide estimates of HCV cases substantially. While the increase in Georgia (by 45 667 cases) would not have doubled the number Rosenberg et al^1^ estimated statewide (56 800), the change was greater than the 0.4% found in their previous sensitivity analysis.^1^ Estimates of statewide prevalence of HCV may improve with accounting for the state’s imprisonment rate and HCV viremia prevalence in the CJ population. Our updates on state seroprevalence diversity are available online.^6^

Limitations in our analysis included considering only 2 states at opposite ends of the HCV prevalence spectrum within CJ populations, at a single time point. While NMCD had the highest seroprevalence in the last survey, it was not an outlier: the prevalence of HCV in the Ohio prison population was 91% that of NMCD.^4^ The Georgia Department of Corrections tied for seventh lowest HCV prevalence.

A revised model using state-specific, observed values of viremia in prisons may help improve the accuracy of future estimates of how many persons in all sectors statewide have HCV infection.
